# Promoting Mental Health and Preventing Mental Illness in General Practice

**DOI:** 10.1080/17571472.2015.1135659

**Published:** 2016-02-24

**Authors:** Steve Thomas, Rachel Jenkins, Tony Burch, Laura Calamos Nasir, Brian Fisher, Gina Giotaki, Shamini Gnani, Lise Hertel, Marina Marks, Nigel Mathers, Catherine Millington-Sanders, David Morris, Baljeet Ruprah-Shah, Kurt Stange, Paul Thomas, Robert White, Fiona Wright

**Affiliations:** ^a^ Mental Health/Learning Disabilities/Dementia Portfolio, NHS Sheffield Clinical Commissioning Group; ^b^ ETHICS Board of Trustees, Epidemiology and Mental Health Policy, Kings College London; ^c^ Primary Care Advisor, Health Education, NW London; ^d^ University of North Carolina, Chapel Hill; ^e^ PAERS Ltd, Health Empowerment Leverage Project; ^f^ Laboratory for Geocultural Analyses (GEOLAB), Ionian University; ^g^ Imperial College, London; ^h^ Strategic Clinical Network for Mental Health, London; ^i^ Educational Trust for Health Improvement through Cognitive Strategies (ETHICS); ^j^ Royal College of General Practitioners; ^k^ Marie Curie National Clinical End of Life Care Champion; ^l^ Mental Health, Inclusion and Community, University of Central Lancashire; ^m^ Primary Care Commissioner and Development Consultant; ^n^ Family Medicine and Community Health, Epidemiology and Biostatistics, Oncology and Sociology, Case Western Reserve University, Cleveland, OH, USA; ^o^ London Journal of Primary Care; ^p^ Ealing Primary Care Mental Health Service, West London Mental Health Trust; ^q^ Public Health Barking and Dagenham Council and Greater London Authority

**Keywords:** Mental health promotion, general practice, mental health integration, public health, parity

## Abstract

This paper calls for the routine integration of mental health promotion and prevention into UK General Practice in order to reduce the burden of mental and physical disorders and the ensuing pressure on General Practice. The proposals & the resulting document (https://ethicscharity.files.wordpress.com/2015/09/rcgp_keymsg_150925_v5.pdf) arise from an expert ‘Think Tank’ convened by the London Journal of Primary Care, Educational Trust for Health Improvement through Cognitive Strategies (ETHICS Foundation) and the Royal College of General Practitioners. It makes 12 recommendations for General Practice: (1) Mental health promotion and prevention are too important to wait. (2) Work with your community to map risk factors, resources and assets. (3) Good health care, medicine and best practice are biopsychosocial rather than purely physical. (4) Integrate mental health promotion and prevention into your daily work. (5) Boost resilience in your community through approaches such as community development. (6) Identify people at increased risk of mental disorder for support and screening. (7) Support early intervention for people of all ages with signs of illness. (8) Maintain your biopsychosocial skills. (9) Ensure good communication, interdisciplinary team working and inter-sectoral working with other staff, teams and agencies. (10) Lead by example, taking action to promote the resilience of the general practice workforce. (11) Ensure mental health is appropriately included in the strategic agenda for your ‘cluster’ of General Practices, at the Clinical Commissioning Groups, and the Health and Wellbeing Board. (12) Be aware of national mental health strategies and localise them, including action to destigmatise mental illness within the context of community development.

## Key messages

• General Practices can integrate mental health promotion and prevention into daily work, by being skilled at biopsychosocial approaches, adapting national mental health strategies to the local context and ensuring good communication and mutual learning between all involved.
• Clusters of General Practices can work with other local organisations to map risk factors, resources and assets within the area and facilitate collaborative community health improvement projects.
• Individual health practitioners can lead by example – promoting their own personal resilience and well-being, and that of their colleagues.
• General practices should support early intervention for people of all ages with signs of illness.

## Why this matters to us

Health promotion and prevention are particularly significant in relation to mental illness, as even optimal treatment at optimal coverage is only able to reduce the burden of mental illness by 28%.[1]

Between 25 and 50% of adult mental illness may be prevented through early intervention in childhood and adolescence.[2] The economic benefits of early childhood interventions have been estimated on average to exceed their costs by a ratio of 1:6.[3]

Many of us on this Think Tank are clinicians who work in General Practice settings. Mental health problems are common in people with medical conditions [4, 5] and develop because of a complex interplay between psychological, physical and social factors. Detection rates are low, so many people do not receive appropriate treatment for their mental health problems.

Our daily work as practitioners makes us recognise the need to improve mental health promotion and prevention in primary care.

For patients, better mental health promotion will improve their quality of life, reduce illness (both mental and physical) and will save lives. In the long run, it will reduce health care use in general practice leading to savings to the NHS and wider society.

Improving mental health promotion and prevention requires a better balance between reactive and anticipatory care. It will require a new balance of activity in General Practice – one that orchestrates and plans more, that anticipates more and reacts less. We recognise that General Practice of the future needs to work in strategic partnerships for whole system improvements, and the skills and models to do this have barely been developed. We hope that this work helps to point the way.

It is also recognised that by improving our own resilience and mental health as clinicians and staff working in the NHS, we will be better able to deal with the complexities that are encountered in daily work and provide good role models to others about how to work well with others.

## Introduction

Good mental health is important for the educational achievement of children and their future prospects, for the physical health of the population, for the social capital (amount of trust and reciprocity) of communities and for the economy. Nonetheless, despite increased efforts to improve mental health treatment services, it remains the case that 10% of children [6] and 17.6% of adults [7] in the UK have a mental disorder at any one time.

Mental disorders in the UK cost around £105 billion a year.[8] Mental disorders are the leading cause of sickness absence in UK, leading to 70 million sick days lost per year. 44% of employment and support allowance benefit claimants report a mental disorder as the primary diagnosis. The cost of crime by those who had conduct problems in childhood is £60 billion in England and Wales.[9]

The proposals in this paper arise from an expert ‘Think Tank’ convened the by the London Journal of Primary Care, ETHICS (http://ethicsfoundation.org/about-ethics/) (Educational Trust for Health Improvement through Cognitive Strategies) and the Royal College of General Practitioners. The Think Tank included professionals from general practice, primary care, public health, psychiatry, community development and commissioning. Participants had expertise in qualitative and quantitative research, epidemiology, guideline development, education and training, primary care development, inter-sectoral collaboration, situation appraisal and policy development.

The group convened in 2015 – twice in February and then March, a week in April and a final meeting in July. Initial outputs can be downloaded from the ETHICS website (http://ethicsfoundation.org/2015/09/04/mental-health-promotion-saves-lives/).The recommendations presented here were launched at the Royal College of General Practitioners in October 2015.

Core principles underpinning this work include the WHO definitions (http://www.who.int/trade/glossary/story046/en/) of health and mental health (http://www.who.int/features/factfiles/mental_health/en/), the Public Health Framework (https://www.gov.uk/government/uploads/system/uploads/attachment_data/file/263658/2901502_PHOF_Improving_Outcomes_PT1A_v1_1.pdf) for health promotion and prevention, the need for parity (equality of time, energy, resources and commissioning expertise) between mental and physical health and the value of a biopsychosocial approach to health.

## Twelve recommendations

### 1. Mental health promotion and prevention are too important to wait

There are compelling reasons to implement mental health promotion and prevention in the GP setting. It will not only reduce illness, save lives and save money, but it will also ultimately reduce the general practice workload as well as promoting resilience and good mental health in the health care workforce.

The UK population is living longer and increasingly with long-term physical health conditions (LTC). It is known that one in three people with a LTC also have a mental health problem.[10]

Depression is more than seven times more common in people with two or more LTCs.[11]

It is also known that up to 75% of lifelong mental ill health will manifest itself by the time a young person reaches 20 years old.[12]

Despite increased efforts to improve mental health treatment services, it remains the case that 10% of children and 17.6% of adults have a mental disorder at any one time. Mental disorders in the UK cost around £105 billion a year.(http://www.centreformentalhealth.org.uk/economic-and-social-costs)

Half of common mental disorders, mostly depression and anxiety remain undiagnosed, and only a quarter receive treatment (http://cep.lse.ac.uk/pubs/download/special/cepsp26.pdf, http://www.hscic.gov.uk/catalogue/PUB02931/adul-psyc-morb-res-hou-sur-eng-2007-rep.pdf). Mental health problems must be actively considered in the management of long-term physical conditions.

Mental disorders are the leading cause of sickness absence in UK, leading to 70 million sick days lost per year (https://www.gov.uk/government/news/cmos-annual-report-employment-is-good-for-mental-health). Indeed, 44% of employment and support allowance benefit claimants report a mental disorder as the primary diagnosis. The cost of crime by those who had conduct problems in childhood is £60 billion in England and Wales. Sainsbury Centre for Mental Health (SCMH) (2009) The chance of a lifetime. Preventing early conduct problems and reducing crime (http://www.ohrn.nhs.uk/resource/policy/SCMHThechanceofalifetime.pdf).

### 2. Work with your community to map risk factors, resources and assets

General Practices can work within GP Federations or developing associations or clusters, Clinical Commissioning Groups, local authorities, Public Health, Health and Wellbeing Boards and other organisations. These should be geographic areas that are small enough to feel you belong and large enough to have political & population effect can become case studies of integrated care.

With Public Health leadership, this partnership of organisations can map local services – health, social care, education, youth and criminal justice services, non-governmental and voluntary sector community organisations. They can map care pathways and services for physical illnesses such as diabetes, mental ill-health such as depression, and social illnesses such as debt, housing and relationship breakdown.

To help target health promotion and evaluate their combined effects, these organisations can amalgamate data on mortality, morbidity, risk factors, income disparity, debt, marital breakdown, substance abuse and resilience. They can support community appraisals to provide sociodemographic information – age structure, employment, ethnicity and language.

General Practices can develop relationships with agencies and signpost to them, for example Stepchange (http://www.stepchange.org) Age UK (http://www.ageuk.org.uk), parenting classes, exercise and dance classes, language classes. They can support partner agencies in their initiatives to improve mental health, for example the ‘whole school approach’ (http://www.fph.org.uk/school_mental_health_promotion).

Practices can analyse their own records to identify vulnerable groups such as looked after children, ex-prisoners, older isolated people and teenage gang members (http://www.familylives.org.uk/advice/teenagers/behaviour/gangs/), asylum seekers and migrants. Other vulnerable groups include members of BME and LGBT communities and those with a learning disability. Black and minority ethnic groups are at two- to threefold increased risk of suicide [14] and a nearly fourfold increased risk of psychosis.[15]

People with learning disability have a twofold increased risk of depression and a threefold increased risk of schizophrenia.[16]

Practices can use their waiting rooms as places where people can learn, using notice boards and videos. Patient participation groups could help patients to use their times profitably when waiting for a consultation. They can link with local libraries and other advocacy services to provide synchrony of effort.

### 3. Good health care, medicine and best practice are biopsychosocial rather than purely physical

A holistic approach is needed if good outcomes are to be achieved. Primary care needs to use a proactive biopsychosocial approach (considering health of someone’s body, mind and relationships) in consultations to assess and manage both mental and physical disorders. They need to use sequences of consultations, the whole team, care plans and patient self-care to address many issues at the same time. Practice staff need to be aware of the common ways in which people with depression and anxiety present, for example sick note requests, fatigue, back pain, headache, mood change, life transition, long-term conditions and attendance by someone who rarely visits the practice.

Primary care practitioners need to be aware of social determinants of ill-health – e.g. debt, unemployment, housing problems, marital problems, alcohol, tobacco and drug misuse.

Once identified, these can either be addressed by direct action where feasible, or frequently by signposting to other relevant agencies and by inking with community development opportunities.[17]

### 4. Integrate mental health promotion and prevention into your daily work

All clinicians need to be able to recognise teachable and learning moments to work with our patients to promote mental health and prevent illness. Such opportunities arise at new patient checks, call and recall systems, immunisation consultations, as well as in routine consultations. Opportunities for mental health promotion and resilience building are present when clinicians are caring for people and families at life transitions, with chronic health conditions and during pre-natal and post-natal care.

Clinicians and managers need to ensure that the practice environment promotes good mental health, using posters, videos and fliers to signpost local services and community assets. Working with local community development workers & third sector organisations, teams inside neighbouring practice need to amalgamate information of resources.

The practice can signpost websites and apps that help patients to help themselves, for example, Best Beginnings (http://www.bestbeginnings.org.uk/baby-buddy-phone-app-parentstobe) for the care of new born babies and the baby buddy app, Emma’s diary (http://www.emmasdiary.co.uk), grandparent plus (http://www.grandparentsplus.org.uk) and the Essential Parent Company (http://essentialparent.com).

Practices can audit their notes to find patients with risk factors for mental disorder. IT clinical reminders can ‘flag’ the severity and range of individual risk factors in each patient. Practice leads for mental health can routinely audit practice rates of severe mental illness, autism, learning disabilities, drinking above safe limits and those at risk of self-harm.

### 5. Boost resilience in your community

Practices can encourage and advertise mental health promotion and prevention for everyone, including physical exercise, good nutrition, use of green space, volunteering and giving, good parenting and social networks.

Social networks and social participation are protective factors against cognitive decline and dementia. Social networks are consistently and positively associated with reduced morbidity and mortality. The Five Ways to Wellbeing (http://www.neweconomics.org/projects/entry/five-ways-to-well-being) are a set of evidence-based actions which promote people’s well-being developed by the New Economics Foundation. They are: *Connect*, *Be Active*, *Take Notice*, *Keep Learning* and *Give*.

Social networks can be improved by community development.[13] This can be funded, supported or led by Clinical Commissioning Groups, local authorities and Trusts in partnership with voluntary organisation. This enhances population resilience and enables organisations to be responsive to the requirements of local populations. This approach enables communities and statutory services to map local assets and harness them for health gain.

### 6. Identify people at increased risk of mental disorder for support and screening

Screen, assess and start prompt treatment and support people who are at increased risk for mental health problems. Practices should take a proactive approach across the life course, knowing the risk and resilience factors for their registered population, at each stage of the life course raising the awareness among Practice staff of mental health need.

Practices should familiarise staff with the factors associated with higher rates of mental disorder among children which include: child abuse; bullying, violence and witnessed violence, institutional care in childhood; physical health problems; poor nutrition; special educational needs; lone parenthood; reconstituted families; poor educational levels; unemployment; low income; psychological distress among mothers and family discord; poor parental mental health; separation of parents; parents in trouble with the police; illegal drug use; deprivation and lack of social cohesion; social and economic upheaval. The WHO publication of 2014 ‘Social Determinants of mental Health’ [18] clearly highlights these factors along the life course along with relevant actions and possible interventions.

Practices should use the opportunities provided by new patient registrations, nurse long-term condition management checks as well as routine consultations, including ante-natal and post-natal care.

A useful document from the Children’s Commissioner ‘Nobody made the connection: the prevalence of neurodisability in young people who offend’ [19] indicates that many young people in custody may have undiagnosed neurodevelopmental disabilities which contributed to the behaviours that led them to offend. The document goes on to assert that assessment, recognition and treatment of neurodevelopmental disorders in children when they are still very young would have significant benefits, allowing the affected children to be diverted from a potential trajectory into the criminal justice system.

Factors associated with higher rates of mental disorder among adults include: being female, aged between 35 and 54, Social class V, tenants of Local Authorities and Housing Associations, separation or divorce, living as a one person family unit, or as a lone parent; debt; IQ of 70–85, no formal educational qualification, people with long-term physical illness, disability, painful and life-threatening conditions.

Consider different settings where groups at high risk may be found. Examples would be high risk occupational groups, including the GP workforce; high risk housing groups including people who are homeless, those in local authority housing, prisoners, people in old people’s homes and looked after children, especially those in institutional care and those at certain life stages, including post-natal women.[20]

Most people who kill themselves have recently seen their GP. Suicide prevention includes the assessment and management of suicidal risk and prompt treatment of underlying depression or other psychological illness. Interventions to promote mental health and resilience, improving coping skills, social networks and social support are necessary. Advice to patients and families about restricted access to lethal medicines including paracetamol, intensive support after previous suicide attempt (there is a 100× increased risk in the following year) and support for high risk occupational groups especially at times of increased risk is essential.

The information about risk factors and risk settings gives the ability to target prevention activities. Ensure that all Practice staff receive regular skills based updates in assessment and management of suicidal risk, in much the same way as we would be done for Child Protection or Cardiopulmonary Resuscitation. Examples of training available: Applied Suicide Intervention Skills Training (https://www.livingworks.net/programs/asist/) or Papyrus (https://www.papyrus-uk.org/training) and Mental Health First Aid (http://mhfaengland.org).

### 7. Support early intervention for people of all ages with signs or symptoms of illness early recognition and intervention is crucial

Early intervention for mental health problems is useful [21]:

• Early intervention for Attention Deficit Hyperactivity Disorder results in improved educational and social outcomes and reduced difficulties in later life.
• Individual parenting intervention programmes for conduct disorder result in improved child behaviour, improved family relationships, improved educational outcomes and reduced conduct disorder, antisocial behaviour and crime.
• School-based intervention programmes for children at highest risk and those with sub-threshold disorders result in improved mental health, improved behaviour at school and home and improved social skills and academic skills.
• Early intervention for depression and anxiety results in less impact on relationships, family and workplace.
• Early intervention for psychosis results in fewer psychotic symptoms, a better course of illness, higher employment rates, reduced GP consultations and Accident and Emergency consultations.
• Early intervention for antisocial personality disorder results in improved functioning for adults, reduced psychopathy and suicidal behaviour.
• Early intervention for harmful drinking results in improved physical and mental health and social functioning.

### 8. Maintain your biopsychosocial skills

Clinicians are responsible for the whole person, not just their physical health, and need to be able to conduct systematic routine assessment of current mental state and suicidal risk in consultations when indicated.

Clinicians need to be skilled, confident and comfortable in dealing with mental illness. They need to be skilled at assessing the severity of depression and severity of suicidal risk. The severity of depression (range of symptoms, severity, frequency, chronicity and interference with daily life) should be assessed. If depression is present, suicidal risk should be assessed and appropriately managed.

### 9. Ensure good communication, interdisciplinary team working and inter-sectoral working with other staff, teams and agencies

Multiagency collaboration and inter-sectoral interventions with local authorities (e.g. public health, social care and education colleagues, health visiting services) and other key sectors is crucial. Examples include the whole school approach to mental health promotion which works on the classroom environment. The provision of parenting classes both in schools and for adults; mental health first aid training for all; action on personal debt; action on social networks for lonely and isolated older people; and suicide risk reduction.

Nursing care across the whole of the life course should support mental health promotion*.* Prevention, treatment, rehabilitation and prevention of mortality need to be organised for the patient in the context of their family and community, whether in or out of hospital. Practice Nurses see most patients with long-term conditions and are well-placed to identify mental health problems. However, most nurses in a GP setting have had no training in recognising or dealing with mental illness. There are many training opportunities, such as those provided by UCLPartners (http://www.uclpartners.com/our-work/academic-health-science-network/integrated-mental-health/practice-nurse-masterclasses) to help address this gap.

Use communication solutions for fast movement of patient information between services and promote IT systems that support interactions between primary and secondary care. Patient online record access & patient access to useful websites e.g. Big White Wall (https://www.bigwhitewall.com/landing-pages/landingv3.aspx?ReturnUrl=/#.Vmrmm4TLPs9), Buddy (https://www.buddyapp.co.uk) and data sharing within practices, between practices, across Clinical Commissioning Groups, between primary and secondary care and local authority public health planning programmes is essential.

### 10. Lead by example, taking action to promote the resilience of the general practice workforce

Consider your own physical and mental health promotion and self-care as health professionals. Conduct reflective practice and support a quality culture within your workplace practice and daily life. For example practitioners need to:

• Learn ways to encourage, experience and model a positive climate in the workplace and a balanced lifestyle; including a staff environment which supports healthy diet, exercise, family time and allows time to give positive feedback and celebration of successes.
• Have protected time to engage in local community development, serve on representative committees, attend Clinical Commissioning Groups and other board meetings, contribute to leadership teams and promote positive mental health and well-being.
• Interact with media and professional groups to raise awareness of general practice staff health needs.
• Encourage an environment where professionals can flourish, where there are opportunities for personal and professional growth, under supportive and inspirational leadership and an enabling environment that supports shared learning for all.
• Encourage an environment where health professionals who experience bereavement or trauma have to face a life changing diagnosis, or are otherwise overwhelmed by multiple stresses can develop resilience and return to work when recovered.
• Encourage an environment where practice staff plan appropriately for retirement.

### 11. Ensure mental health is appropriately included in the strategic agenda for your cluster, at the Clinical Commissioning Groups, and the Health and Wellbeing Board

Health care policy needs to ensure parity between mental and physical health*,* including equality of time, energy, resources and commissioning expertise.

Mental health promotion and prevention needs to be included in all commissioning contracts and in all service provision*.* For example, by the inclusion of evidence-based interventions such as parenting classes, a whole school approach to mental health promotion, Mental Health First Aid training, addressing social risk factors such as debt, bullying and child abuse.

Integrate mental health promotion into all care pathways for people with physical and/or mental illness. The document ‘The Future’s Digital: Mental Health and Technology’ (http://www.nhsconfed.org/~/media/Confederation/Files/Publications/Documents/the-futures-digital.pdf) gives helpful ideas about how the use of digital technology can potentially improve mental health outcomes and be used to promote good mental health.

### 12. Be aware of national mental health strategies and localise them, especially action to destigmatise mental illness

We need to talk about mental health and mental illness as every day, important and normal issues – as normal as going to work or having asthma. For example, Time to Change (http://www.time-to-change.org.uk/timetotalkday) is an anti-stigma campaign run by the mental health charities *Mind* and *Rethink Mental Illness*. The aim is to provide a forum to empower people with mental health problems to talk about issues and to provide high profile marketing and media campaigns.

## Disclosure statement

No potential conflict of interest was reported by the authors.
